# Neuroendocrine cell hyperplasia of infancy: an unusual cause of hypoxemia in children

**DOI:** 10.1186/s13052-016-0295-y

**Published:** 2016-09-15

**Authors:** Silvia Caimmi, Amelia Licari, Davide Caimmi, Anna Rispoli, Eugenio Baraldi, Fiorella Calabrese, Gian Luigi Marseglia

**Affiliations:** 1 Department of Pediatrics, University of Pavia, Fondazione IRCCS Policlinico San Matteo, Pavia, Italy; 2 Unité d’allergologie, Département de Pneumologie et Addictologie, Hôpital Arnaud de Villeneuve, CHU de Montpellier, Montpellier, France; 3 Sorbonne Universités, UPMC Paris 06, UMR-S 1136, IPLESP, Equipe EPAR, F-75013 Paris, France; 4 Department of Radiology, Fondazione IRCCS Policlinico San Matteo, Pavia, Italy; 5 Women’s and Children’s Health Department, Unit of Respiratory Medicine and Allergy, University of Padova, Padova, Italy; 6 Department of Cardiothoracic and Vascular Sciences, Pathological Anatomy Section, University of Padova, Padova, Italy

**Keywords:** Neuroendocrine cell hyperplasia, Hypoxemia, Interstitial lung disease in children, Persistent tachypnea, Ground-glass opacification

## Abstract

**Background:**

Childhood interstitial lung disease (chILD) is a heterogeneous group of rare disorders characterized by abnormal imaging findings, impaired gas exchange; and is associated with substantial morbidity and mortality. Neuroendocrine cell hyperplasia (NEHI) is a unique sub-group, which is more prevalent in infants and children younger than 2 years of age, and typically manifests with chronic tachypnea, retractions, hypoxemia and failure to thrive. NEHI insidiously appears in the first year of life, subtly masquerading as one of the more common lung diseases of childhood. Therefore, the diagnosis of NEHI is challenging and requires a systematic approach.

**Case presentation:**

We report a case of an infant, with a history of recurrent respiratory infections and wheezing, who presented with persistent hypoxemia (PaO2 88 mmHg) and chronic respiratory symptoms, that prompted an extensive diagnostic work up for chILD; eventually a diagnosis of NEHI was made.

**Conclusion:**

NEHI is a rare chILD disorder presenting in the first 2 years of life with common but challenging key clinical features. Increased awareness among pediatricians and prompt recognition of the clinical presentation may enable timely diagnosis and improve disease management and prognosis.

## Background

Childhood interstitial lung disease (chILD) is a heterogeneous group of rare disorders characterized by abnormal imaging findings, impaired gas exchange; and is associated with substantial morbidity and mortality. [1]. A recent revised classification identified neuroendocrine cell hyperplasia (NEHI) as a prevalent sub-type in infants and children younger than 2 years of age [2, 3]. The chILD clinical phenotype refers to children who meet at least three of the following four criteria: (1) respiratory symptoms (cough, tachypnea, or exercise intolerance), (2) signs (resting tachypnea, adventitious sounds, retractions, digital clubbing, failure to thrive or respiratory failure), (3) hypoxemia, and (4) specific diffuse abnormalities on chest imaging [1]. In infancy, the clinical features of chILD may be non-specific, occasionally subtle and masquerade as more common lung diseases in childhood (eg, cystic fibrosis, immunodeficiency, or congenital heart disease). Therefore, establishing a specific diagnosis of chILD is challenging and requires a systematic approach.

We report a case of an infant, with a history of recurrent respiratory infections and wheezing, who presented with persistent hypoxemia (PaO2 88 mmHg) and chronic respiratory symptoms, that prompted an extensive diagnostic work up for chILD; eventually a diagnosis of NEHI was made.

## Case presentation

The patient, a 5-month-old boy, was delivered full-term and vaginally without complications. The neonatal course was uneventful. At discharge, he was exclusively breastfed and his weight and length were in the 50th percentile.

A family history revealed consanguineous parents coming from Northern Africa; the mother had a history of two miscarriages before the birth of his older siblings. His elder brother had asthma and presented with abnormalities of the rib cage, while his sister underwent surgical treatment for transposition of the great vessels.

At age 3 months, after a chickenpox infection, he experienced his first respiratory exacerbation followed by recurrent wheezing associated with airway infections, that required frequent admissions to the emergency room and treatment with inhaled beta2-adrenergic drugs, antibiotics and oral steroids. The child had received all of the mandatory and recommended vaccines for his age; he had no allergies and no history of apparent prior failure to thrive. At the age of 5 months, he was admitted to our Pediatric Unit for acute respiratory distress in a framework of suspected viral bronchiolitis. He presented with tachypnea (103/min), retractions, and hypoxemia (oxygen saturation on room air at 88 % at rest), requiring oxygen supplementation. Expiratory wheezing and crackling were the main respiratory auscultation findings. A clinical examination also revealed a significant reduction in growth velocity (both weight and length from the 50th to 10th percentile). Blood gas analysis revealed a respiratory acidosis (pH: 7,30; PaCO2: 48 mmHg; HCO3: 19 mmol/l). A chest radiograph revealed over-inflation of the lungs with some interstitial markings in the pulmonary hilum and right upper lobe. Nasal secretions were analyzed for respiratory syncytial virus, rhinovirus, influenza virus, parainfluenza virus and adenovirus; all resulted negative. After a first therapeutic trial with nebulized β_2_-adrenergic agonists without improvement of symptoms, he was treated with respiratory support in combination with appropriate fluid and nutrition management.

**Fig. 1:**
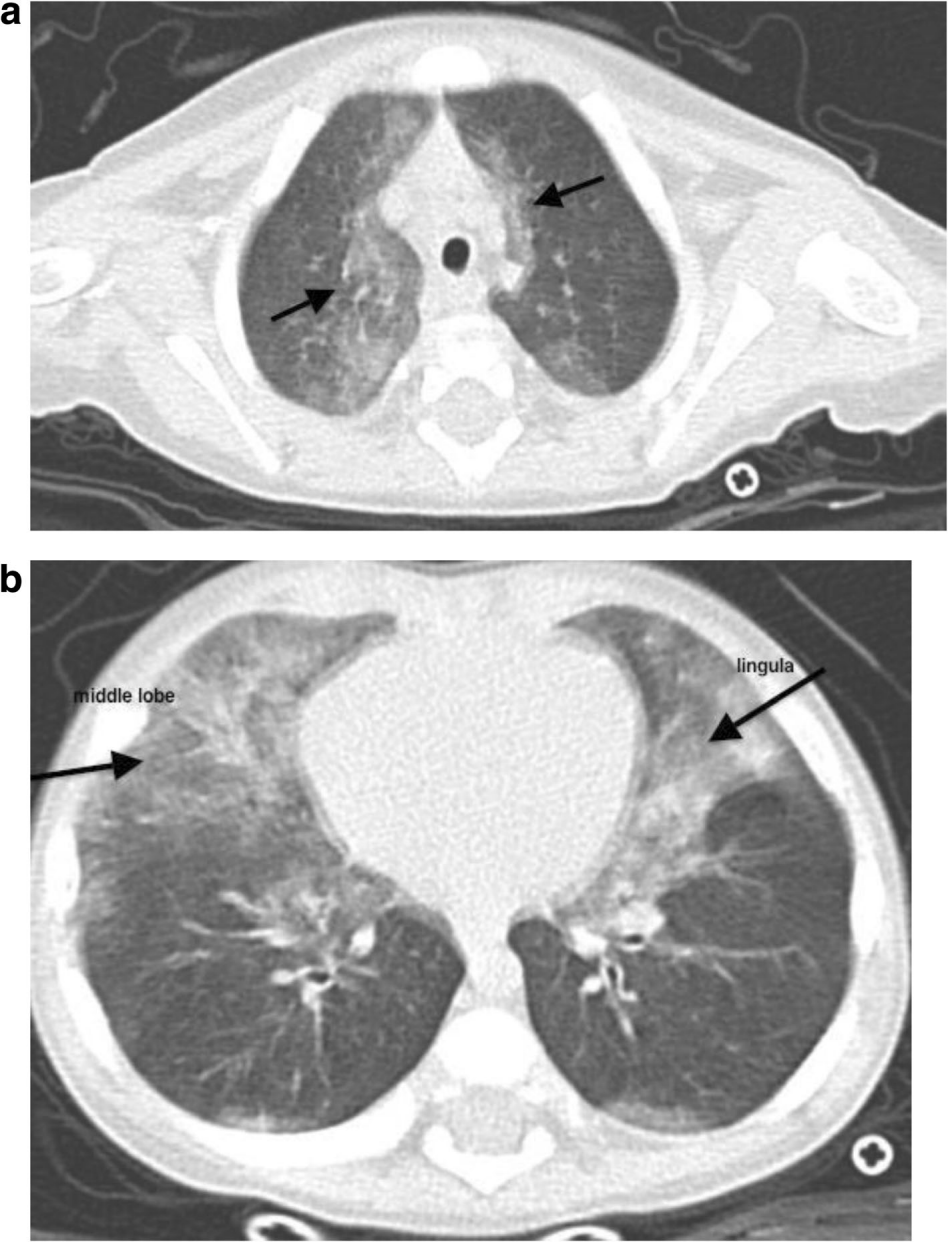
Areas of interest: ground glass opacities in the medial portion of the upper lobes (**a**) and right middle lobe and lingula (**b**) with some areas of consolidation in the lingula

**Fig. 2:**
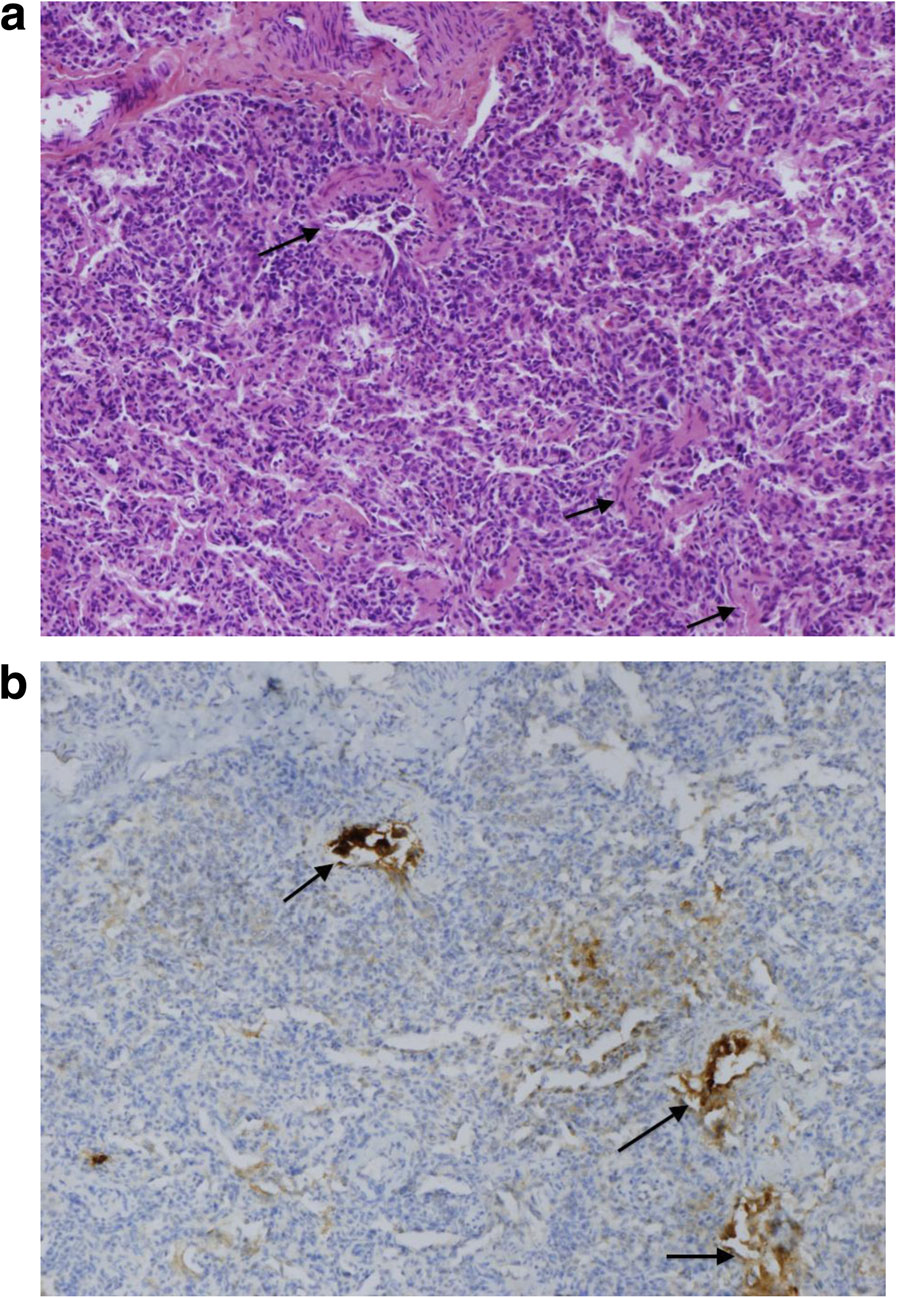
Panoramic view of the lung parenchyma; alveolar structures are normal without any fibrosis; arrows show bronchi with hyperplastic neuroendocrine cells; staining with hematoxylin-eosin, Magnification 100× (**a**); immunostaining with bombesin, Magnification 100× (**b**)

Considering the persistent hypoxemia, despite supplemental oxygen support together with the associated pronounced respiratory distress and the clinical and radiological findings, the bronchiolitis diagnosis was determined to be misleading and an extensive diagnostic workup was performed. Complete blood count and measurement of serum immunoglobulin levels were normal; sweat test, serological tests for bacterial infections (*Chlamydia* and *Mycoplasma pneumoniae*, *Bordetella pertussis*), echocardiography and intraluminal impedance pH monitoring were negative. Assessment of the ciliary ultrastructure, beat frequency and pattern from a brush biopsy of the nasal epithelium excluded the diagnosis of primary ciliary dyskinesia. Subsequently, genetic testing for all exons encoding the ABCA3 surfactant gene (ATP-binding cassette 3) and the SFTPC gene (surfactant protein C) was carried out for suspected genetic surfactant disorders, with negative results. A diagnostic bronchoscopy excluded airway malacia and a bronchoalveolar lavage (BAL) revealed the high prevalence of neutrophils and the presence of slightly enlarged lymphocyte aggregates and macrophages with intracellular lipids. Microbiological examination of the BAL fluid showed an important colonization by *Streptococcus pneumnoniae, Moraxella catarrhalis*, *Rhinovirus* and *Adenovirus*. Meanwhile, high-resolution chest computed tomography (HRCT) showed air trapping in the left upper lobe and both lower lobes, and ground-glass opacities of the middle-upper lobes and of the lingula (Fig. 1). This typical radiological pattern raised the possibility of neuroendocrine cell hyperplasia of infancy (NEHI). In order to complete the diagnostic workup, an open lung biopsy performed at 9 months of age showed the presence of areas characterized by macrophage alveolitis associated with neuroendocrine cell hyperplasia of the small airways. Immunohistochemistry demonstrated a significant number of bombesin-positive cells in the small airways walls (Fig. 2). Finally, NEHI was diagnosed on the basis of radiological and histopathological findings typical of NEHI, while pulmonary interstitial glycogenosis was excluded with the biopsy findings. The infant was initially treated with oral prednisolone (1 mg/kg daily); afterwards, he required long-term oxygen and nutritional supplementation.

At follow up visits he exhibited gradual clinical improvement. At 12 months of age, oxygen support was administered only at night. At the age of 18 months, his respiratory rate improved and his growth values were back within the normal range (50th percentile). At this time, the daily dose of steroids was stopped. Currently, at age 2 years, he only requires symptomatic treatment of any respiratory infection.

## Conclusion

NEHI is a disorder of unknown etiology that typically manifests with chronic tachypnea, retractions, hypoxemia and failure to thrive, insidiously appearing in the first year of life. Crackles are prominent on chest auscultation, while wheezing is uncommon [4, 5]. The incidence and prevalence of NEHI, as well as its etiology, are still unknown. Familial cases with affected siblings have been reported, suggesting that there might be a genetic predisposition [6, 7].

The chest radiograph may be normal or may reveal hyperinflation with variable increased peri-hilar opacity [8], whereas HRCT appears to be the most reliable non-invasive imaging test that can point to the diagnosis of NEHI and can differentiate NEHI from other types of interstitial lung disease [9]. Multi-lobar ground-glass opacity predominantly involving the right middle lobe and lingula as well as a mosaic pattern of air-trapping are typical findings on HRCT with a reported sensitivity of 83 % and specificity of 100 %, thus limiting in some cases the indication for a confirmatory lung biopsy [9]. Whenever available, lung function testing shows a pattern of hyperinflation and airflow obstruction [10, 11]. Bronchoscopy with BAL plays a role in excluding infection, aspiration or haemorrhage, and in identifying pulmonary alveolar proteinosis [12]; but lung biopsy remains the gold standard for chILD when less invasive tests fail to secure a diagnosis [1]. Lung biopsies in NEHI often show an essentially normal histology on standard staining, with an increase in Bombesin-positive cells after specific staining [4, 13]. Nevertheless, it should be noted that hyperplasia of neuroendocrine cells is not specific for NEHI, and has also been described in several other rare pediatric lung diseases [13, 14].

In our report, the patient was initially hospitalized presenting with non-specific features of acute hypoxemia, crackles and tachypnea; his misleading clinical history of recurrent episodes of wheezing associated with respiratory infections led to a first diagnosis of bronchiolitis and resulted in a delayed NEHI diagnosis. Moreover, in his first 5 months of life he did not present with failure to thrive, which is one of the key features in chILD disorders. The persistent hypoxemia and increased respiratory effort raised concerns regarding the initial diagnosis. Additionally, the clinical history of neonatal unexplained tachypnea, the familiar history of thoracic abnormalities, and evidence of a previously unrecognized failure to thrive were all elements that led to the decision to perform an extensive differential diagnostic work up. Overlapping common respiratory disorders that should be considered in the clinical differential work up include infections, asthma, airway anomalies and injury, gastro-esophageal reflux, foreign body aspiration, cystic fibrosis, primary ciliary diskinesia, immunodeficiency and congenital heart disease [1, 12]. In addition, other causes of chILD such as pulmonary hypoplasia, pulmonary interstitial glycogenosis and genetic disorders of surfactant production and metabolism needed to be excluded [1, 15, 16].

There is no consensus on the therapy for NEHI, and management generally consists of supportive care: supplemental oxygen for chronic hypoxemia, adequate nutrition, proper immunization, avoidance of environmental pollutants, and treatment of recurrent infections [4, 12]. In some cases of chILD, steroids may also be used to improve the clinical picture, but with close monitoring of side effects [12].

The long-term outcome of NEHI is generally favourable with most patients gradually improving over time, although persistent airway obstruction mimicking severe asthma and relapse with respiratory infection have also been reported [17]. In our case, it was important to schedule a close follow up of the patient who was at risk of developing severe asthma as well, since he has a family history of asthma, exhibits macrophage alveolitis and has previously undergone systemic steroidal therapy.

In conclusion, NEHI is a rare chILD disorder presenting in the first 2 years of life with common but challenging key clinical features, in particular hypoxemia, respiratory distress and failure to thrive, and distinct imaging and histological findings. Increased awareness among pediatricians and prompt recognition of the clinical presentation may enable timely diagnosis and improve disease management and prognosis.

## Abbreviations

BAL: Bronchoalveolar lavage
chILD: Childhood interstitial lung disease
HRCT: High resolution computed tomography
IPFTs: Infant pulmonary function tests
NEC: Neuroendocrine cell
NEHI: Neuroendocrine cell hyperplasia of infancy
PNECs: Pulmonary neuroendocrine cells
PTI: Persistent tachypnea of infancy
